# Adverse Events of Everolimus in Patients with Tuberous Sclerosis Complex Treated for Renal Angiomyolipoma/Subependymal Giant Cell Astrocytoma

**DOI:** 10.7150/ijms.88022

**Published:** 2023-09-04

**Authors:** Shuo-Yan Gau, Sung-Lang Chen, Cheng-Siu Chang, Teng-Fu Tsao, Jeng-Dau Tsai

**Affiliations:** 1 School of Medicine, Chung Shan Medical University, Taichung, Taiwan.; 2 Institute of Medical Education, Chi Mei Medical Center, Tainan, Taiwan.; 3 Department of Urology, Chung Shan Medical University Hospital, Taichung, Taiwan.; 4 Department of Neurosurgery, Chung Shan Medical University Hospital, Taichung, Taiwan.; 5 Department of Medical Radiology, Chung Shan Medical University Hospital, Taichung, Taiwan.; 6 Department of Pediatrics, Chung Shan Medical University Hospital, Taichung, Taiwan.

## Abstract

**Background:** Although regarded as a potentially efficient approach to address tuberous sclerosis complex (TSC)-associated complications, the adverse event profile of everolimus has not yet been fully elucidated. The present study aimed to clarify the adverse event spectrum in patients with TSC who are using everolimus for common indications, in comparison to those who do not use everolimus.

**Materials and Methods:** We recruited patients with TSC who were followed up annually at TSC integrated clinics or referred for medical assistance. Medical reviews and laboratory investigations were performed at baseline and annually by clinical physicians. The adverse events were assessed as per the National Cancer Institute Common Terminology Criteria for Adverse Events.

**Results:** Common adverse events in everolimus users included hypercholesterolemia (55%), gingivostomatitis (50%), proteinuria (50%), and hyperglycemia (40%). Compared with everolimus nonusers, the occurrence of gingivostomatitis and proteinuria was significantly higher in everolimus users (gingivostomatitis, p=0.02; proteinuria, p=0.02). Among the everolimus users, 12 patients had level I CTCAE, and five had level II CTCAE. None of the everolimus users presented with CTCAE level III or higher.

**Conclusion:** Patients with TSC who are everolimus users had a higher tendency to develop gingivostomatitis and proteinuria compared to nonusers. However, no differences were observed in the occurrence of other adverse events between everolimus users and nonusers.

## Introduction

As an autosomal-dominant disease, tuberous sclerosis complex (TSC) results in symptomatic involvement of various organ systems, including dermatological, renal, and central nervous systems 1, 2. In a recent population-based study from France, the incidence of TSC was reported to be 0.44 per 100,000 person-years 3. The presence of TSC can significantly negatively influence patients' quality of life due to the impairment of the psychological and physical domains 4, 5.

In recent years, as the pathophysiology of gene abnormalities of *TSC1* and *TSC2* and their subsequent influence on the downstream mTOR pathway have been gradually clarified, the therapeutic role of mTOR inhibitors in TSC treatment has also been evaluated 6. It has been reported that everolimus, an mTOR inhibitor, presents significant clinical efficacy in improving subependymal giant cell astrocytoma (SEGA), angiomyolipoma (AML), lymphangioleiomyomatosis (LAM), and facial angiofibroma caused by TSC 7-9.

Considered as a potentially efficient approach to address TSC-associated complications, the adverse event profile of everolimus has been examined under different clinical contexts in large scale studies 10-12. Results of the EXIST-1 and EXIST-2 studies suggested that elevation of serum creatinine and proteinuria were observed as potential adverse events in patients taking everolimus 10. However, to the best of our knowledge, the adverse event profile of everolimus users with TSC in Taiwan is lacking. To bridge the knowledge gap, we conducted a clinical study to clarify the adverse event spectrum in patients with TSC who are using everolimus for common indications, in comparison to those who do not use everolimus.

## Methods and Materials

### Patient selection and study design

**Figure 1:**
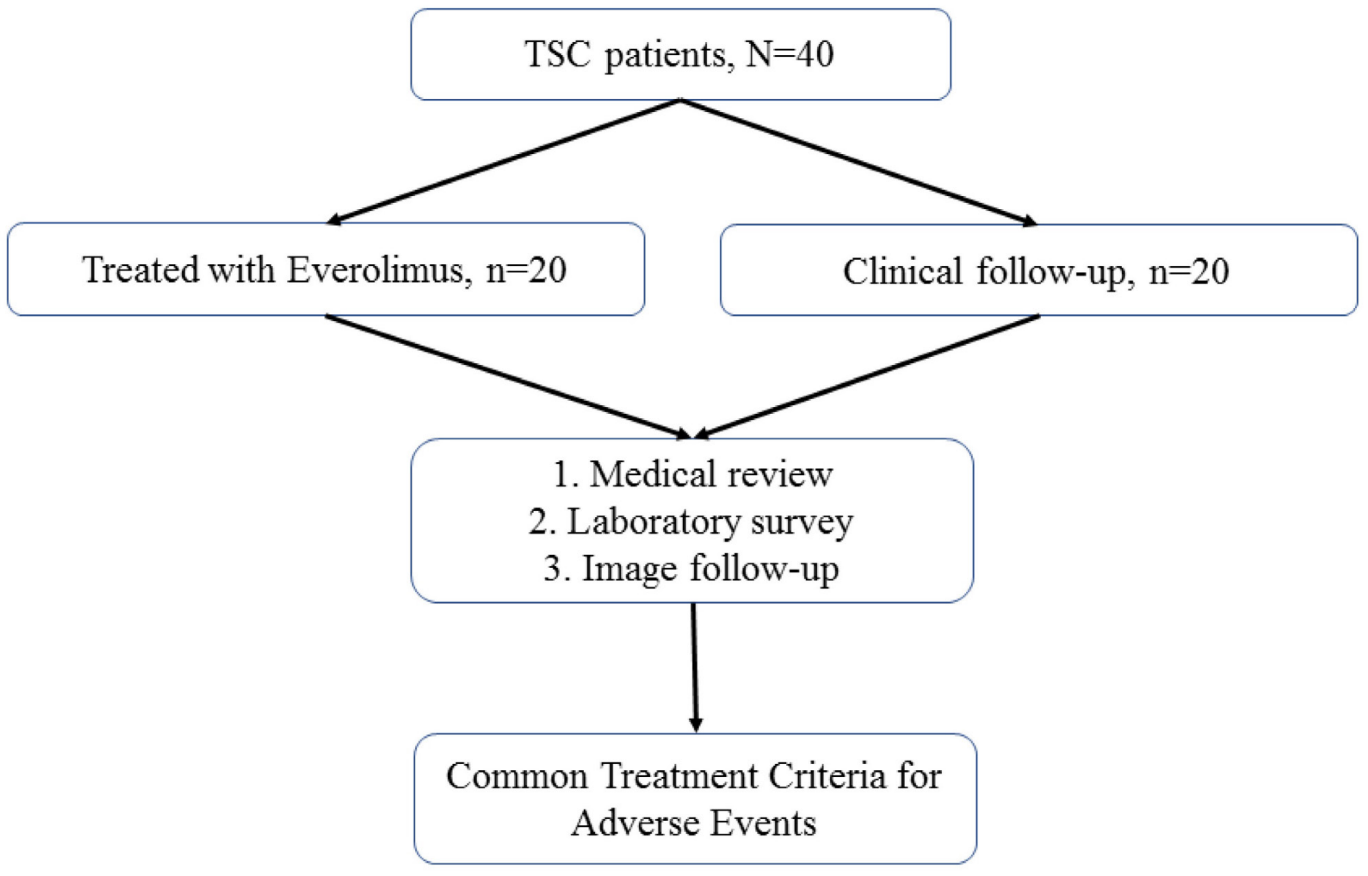
Patient selection flowchart

Patients with TSC were recruited for this study. All patients were followed up annually at TSC-integrated clinics or referred for medical assistance at the three branches of Chung Shan Medical University Hospital, a tertiary center in central Taiwan. The detailed patient selection process is illustrated in **Figure 1**. The diagnostic criteria for TSC were based on the updated International Tuberous Sclerosis Complex Diagnostic Criteria 15. The indications for everolimus included TSC-associated SEGA, AML, and LAM. For each everolimus user, the initial dose of everolimus was 2.5 mg per day for all enrolled patients; this was titrated up to 5.0 mg per day.

### Assessment of adverse effects

Adverse events were assessed as per the National Cancer Institute Common Terminology Criteria for Adverse Events (CTCAE, version .0), which is widely utilized in clinical studies 16. Medical reviews and laboratory investigations were performed at baseline and annually by clinical physicians. To monitor changes in the laboratory data and identify potential adverse events, regular laboratory investigations were conducted. These investigations included routine blood tests, biochemistry profiles, HBV/HCV titers, and routine urinary tests.

### Statistical methods

Categorical variables are presented as frequencies (n) and percentages (%). The chi-squared test was used to compare categorical variables. Statistical significance was defined as p < 0.05. All statistical analyses were conducted using SPSS for Windows (version 26.0; SPSS Inc., Chicago, IL, USA).

### Ethnic Statement

The study was conducted in accordance with The Declaration of Helsinki and approved by the Institutional Review Board of Chung Shan Medical University (CSMUH No.CS1-22076).

## Results

### Baseline characteristics

Among all the enrolled patients with TSC, 20 were treated with everolimus and 20 were not (**Figure 1**). No statistically significant differences in age, sex, or genotype were observed between everolimus users and nonusers (**Table 1**).

### Medical utilization profiles

Among the enrolled everolimus users, the indication for everolimus use was mostly for the treatment of AML (n=12; 60% of everolimus users), whereas approximately 30% of everolimus users were prescribed medication for the treatment of SEGA (n=6). The mean age at administration was 26.6 years, and the mean duration was 5.5 years. The mean daily dose was 4.6 mg, while the mean trough level was 7.6 ng/ml (**Table 2**).

### Adverse events

Adverse events in everolimus users and nonusers are presented in** Table 3**. Common adverse events, including hypercholesterolemia (55%), gingivostomatitis (50%), proteinuria (50%), and hyperglycemia (40%), were observed in everolimus users. Compared with everolimus nonusers, the occurrence of gingivostomatitis and proteinuria was significantly higher in everolimus users (gingivostomatitis, p=0.02; proteinuria, p=0.02). Among the everolimus users, 12 patients had level I CTCAE, and five had level II CTCAE. None of the everolimus users presented with CTCAE level III or above. Among everolimus nonusers, 12 patients were categorized as level I, while two patients were categorized as level II. Similar to the everolimus users, none of the patients in this group presented with CTCAE levels III or IV.

## Discussion

In this study, we reported the adverse event profiles of everolimus users with different indications, including TSC-associated AML, SEGA, and LAM. Most adverse events in the different organ systems did not show statistically significant differences between everolimus users and nonusers. However, incident gingivostomatitis and proteinuria were more frequently observed in the everolimus users than in the nonusers. Considering that studies investigating the adverse event profiles of everolimus are scarce and are generally limited to a small number of participants or lack control groups 13, 14, the results reported in this study could potentially be useful for clinicians when considering management strategies in patients with TSC.

Sirolimus and everolimus are the two most well-known mTOR inhibitors for TSC management; they target mTORC1 and separate its connections with the cofactor FKB12 17. The mTORC1 inhibitor-associated adverse events such as infection, proteinuria, and stomatitis were widely reported in randomized controlled trials and single-arm studies 6, 10, 18. The most common adverse events of mTOR inhibitors, such as stomatitis, were reported to be mild and did not seem to influence the continuation of medication prescriptions 6. However, severe infectious adverse events have been reported in previous randomized controlled trials (EXIST-3 study) among mTOR inhibitor users, resulting in severe pneumonia, septic shock, and subsequent participant deaths 19. In the current study, infectious adverse events, including upper airway respiratory infection, pneumonia, and hepatitis virus B infection, were numerically high in the everolimus group. However, compared with nonusers, the difference in the percentage of infectious events was insignificant. Moreover, no adverse events above CTCAE level III were noted, with most participants presenting with a CTCAE level I (60%). These findings indicated that in Taiwanese patients with TSC who are everolimus users, the severity and categories of adverse events are similar to those reported in previous international studies.

The adverse events associated with everolimus have been widely evaluated in clinical studies. In a recent Dutch study based on electronic medical records, it was reported that in TSC patients, total cholesterol, LDL- and HDL-cholesterol levels at baseline were similar to patients without TSC. However, the use of everolimus could lead to elevation of these lab data 20. In our current study, we report that the occurrence of hypercholesterolemia did not present statistically significant difference between everolimus users and non-users. Another international real-world evidence revealed that 14 of 174 patients with TSC who were everolimus users (7.8%) presented with stomatitis during the follow-up period, whereas 6.1% of the participants presented with hypercholesterolemia 21. In a Japanese clinical study, Hatano et al. reported that more than 25% of patients with TSC prescribed low-dose everolimus (5 mg QD) presented with adverse events including stomatitis, irregular menstruation, and nasopharynxgitis. When comparing the difference in adverse event profiles between low-dose and conventional dosage users (10 mg QD), only the presence of stomatitis was statistically significant; low-dose everolimus users presented with stomatitis less frequently after using everolimus 13. The results of the current study correspond with previous evidence reporting a high prevalence of gingivostomatitis and hypercholesterolemia in patients with TSC who are everolimus users. However, among all evaluated adverse events, only gingivostomatitis and proteinuria in the everolimus group showed significant differences compared with the control group. Further large-scale case-control studies are necessary to compare the prevalence of adverse events in other organ systems, including the endocrine and gastrointestinal systems, between everolimus users and nonusers.

The strength of this study lies in its ability to provide information on the adverse event profiles of everolimus users and compare them with those of everolimus nonusers for common TSC-associated indications. By comparing the adverse event percentage of nonusers, we were able to provide a clearer view of the adverse event profiles in everolimus users, compared with those obtained from single-arm studies. However, there are some limitations to this study that should be considered when interpreting the results. First, considering that this study was conducted at a medical center in central Taiwan, the results may not be generalizable. Second, the number or participants in this study could be too small to show differences in critical parameters such as serum lipids (total cholesterol, LDL and HDL, etc.) between everolimus users and non-users. In this case, we might not be able to perform further analyses regarding these parameters and evaluate their detailed interplay with the use of everolimus. Future large-scale studies are warranted to compare the incidence of adverse events among everolimus users from different populations and ethnicities.

In conclusion, patients with TSC who are everolimus users had a higher tendency to develop gingivostomatitis and proteinuria than nonusers. However, no differences in the occurrence of other adverse events were noted between everolimus users and nonusers. Based on the results of the current study, the safety profile of everolimus should be considered by clinicians when caring for patients with TSC.

### Guarantor of the article

Dr. Jeng-Dau Tsai is the corresponding author who is responsible for the accuracy and integrity of data analysis.

### Specific author contributions

All the authors involved in drafting or revising the article and approved of the submitted version.

Study conception and design: Gau SY, Tsai JD, Chen SL and Tsao TF.

Data acquisition: Gau SY, Tsai JD.

Data analysis and demonstration: Gau SY, Tsai JD.

Original draft preparation: Gau SY, Tsai JD, Chen SL and Tsao TF.

### Statement of ethics

The hospital's Institutional Review Board of Chung Shan Medical University Hospital approved this study (CSMUH No. CS1-22076) and the study was performed according to the Declaration of Helsinki. All study participants were provided of informed consent.

### Data sharing statement

The original data analyzed in the present study can be provided by the authors upon reasonable request.

## Figures and Tables

**Table 1:** Demographic data of TSC patients, n=40

Variable		All	TSC with everolimus		P value
			Yes (group A)	No (group B)	
Numbers		40	20	20	
Age (year, range)		5-54	31.7 (9.7)	21.4 (31.7)	> 0.05
Gender					
	male	13	4	9	> 0.05
	female	29	16	11	
Gene					
	TSC 1	7	4	3	> 0.05
	TSC 2	21	9	12	
	NMI/ND	12	7	5	

NMI: no mutation identified; ND: not done. NS: not significant.

**Table 2:** Indications and administration profile of TSC patients treated with everolimus, n=20

Indication			Patient amount	Percentage (%)
		AML	12	60
		SEGA	6	30
		LAM	2	10
**Administration**			**Mean**	**SD**
	Age of administration (years old)		26.6	9.5
	Duration of administration (year)		5.5	3.2
	Daily dose (mg)		4.6	0.9
	Mean trough levels (ng/ml)		7.6	5.7
				

AML, angiomyolipoma; SEGA, subependymal giant cell astrocytoma; LAM, lymphangioleiomyomatosis; SD, standard difference

**Table 3:** Adverse events of TSC patients with or without administration of everolimus

Variable		Administration of everolimus (%)		P value
		Yes (n=20)	No (n=20)	
Immune system disorder				
	Gingivostomatitis	10 (50)	3 (15)	**0.02**
Skin disorder				
	Acnes	7 (35)	9 (45)	0.37
Infection disorder				
	URI	3 (15)	1 (5)	0.30
	Pneumonia	4 (20)	1 (5)	0.17
	HBV	4 (20)	3 (15)	0.50
	HCV	0 (0)	0 (0)	-
Gastrointestinal disorders				
	Diarrhea	4 (20)	1 (5)	0.17
Endocrine disorder				
	Amenorrhea	4 (20)	0 (0)	0.10
Metabolic disorder				
	Hypertriglyceridemia	5 (25)	4 (20)	0.50
	Hypercholesterolemia	11 (55)	6 (30)	0.10
	DM	3 (15)	1 (5)	0.30
	Hyperglycemia	8 (40)	5 (25)	0.25
Renal and urinary disorder				
	UTI	1 (5)	0 (0)	
	Pyuria	4 (20)	3 (15)	0.50
	Hematuria	6 (30)	2 (10)	0.77
	Proteinuria	10 (50)	3 (15)	**0.02**
CTCAE				
	0	3 (15)	6 (30)	
	I	12 (60)	12 (60)	
	II	5 (25)	2 (10)	
	III	0 (0)	0 (0)	
	IV	0 (0)	0 (0)	

URI: upper airway respiratory infection; UTI: urinary tract infection; HPF, high-powered field; CTCAE: Common Terminology Criteria for Adverse Events
